# (*E*)-*N*′-(3-Bromo-5-chloro-2-hy­droxy­benzyl­idene)nicotinohydrazide

**DOI:** 10.1107/S1600536811038268

**Published:** 2011-09-30

**Authors:** M. Prabhu, K. Parthipan, A. Ramu, G. Chakkaravarthi, G. Rajagopal

**Affiliations:** a Shasun Pharmaceuticals Ltd, Chennai 600 048, India; b Department of Chemistry, Pondicherry University, Pondicherry 605014, India; c Department of Inorganic Chemistry, Madurai Kamaraj University, Madurai 625 021, India; d Department of Physics, CPCL Polytechnic College, Chennai 600 068, India; e Department of Chemistry, Government Arts College, Melur 625 106, India

## Abstract

There are two independent mol­ecules in the asymmetric unit of the title compound, C_13_H_9_BrClN_3_O_2_, in which the dihedral angles between the benzene and pyridine rings are 8.23 (9)° and 52.84 (12)°. Both the mol­ecules exist in an *E* configuration with respect to the C=N double bond. The two mol­ecules in the asymmetric unit are linked *via* weak C—H⋯O hydrogen bonds. In both the mol­ecules, an intra­molecular O—H⋯N hydrogen bond generate an *S*(6) graph-set motif. In the crystal, inter­molecular N—H⋯O and C—H⋯O hydrogen bonds generate bifurcated *R* ^1^_2_(7) ring motifs. The crystal packing is further stabilized by weak inter­molecular N—H⋯O, N—H⋯N, C—H⋯O and π–π [centroid–centroid distance 3.615 (2) Å] inter­actions.

## Related literature

For related structures, see: Naveenkumar *et al.* (2010 ▶); Su *et al.* (2010 ▶); Tecer *et al.* (2010 ▶). For hydrogen-bond motifs, see: Bernstein *et al.* (1995 ▶).
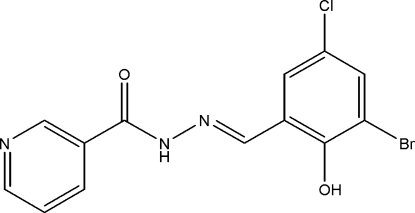


## Experimental

### 

#### Crystal data

C_13_H_9_BrClN_3_O_2_
*M* *_r_* = 354.59
Monoclinic, 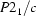

*a* = 18.2217 (5) Å
*b* = 7.4666 (2) Å
*c* = 23.6916 (5) Å
β = 122.685 (1)°
*V* = 2712.93 (12) Å^3^
*Z* = 8
Mo *K*α radiation
μ = 3.23 mm^−1^
*T* = 295 K
0.30 × 0.24 × 0.20 mm

#### Data collection

Bruker APEXII diffractometer
Absorption correction: multi-scan (*SADABS*; Sheldrick, 1996 ▶) *T*_min_ = 0.444, *T*_max_ = 0.564
34991 measured reflections
8116 independent reflections
4715 reflections with *I* > 2σ(*I*)
*R*_int_ = 0.042

#### Refinement

*R*[*F* ^2^ > 2σ(*F* ^2^)] = 0.047
*wR*(*F* ^2^) = 0.125
*S* = 1.02
8116 reflections
377 parameters
4 restraints
H atoms treated by a mixture of independent and constrained refinement
Δρ_max_ = 1.27 e Å^−3^
Δρ_min_ = −0.84 e Å^−3^

### 

Data collection: *APEX2* (Bruker, 2004 ▶); cell refinement: *SAINT* (Bruker, 2004 ▶); data reduction: *SAINT*; program(s) used to solve structure: *SHELXS97* (Sheldrick, 2008 ▶); program(s) used to refine structure: *SHELXL97* (Sheldrick, 2008 ▶); molecular graphics: *PLATON* (Spek, 2009 ▶); software used to prepare material for publication: *SHELXL97*.

## Supplementary Material

Crystal structure: contains datablock(s) global, I. DOI: 10.1107/S1600536811038268/ng5231sup1.cif

Structure factors: contains datablock(s) I. DOI: 10.1107/S1600536811038268/ng5231Isup2.hkl

Supplementary material file. DOI: 10.1107/S1600536811038268/ng5231Isup3.cml

Additional supplementary materials:  crystallographic information; 3D view; checkCIF report

## Figures and Tables

**Table 1:** Hydrogen-bond geometry (Å, °)

*D*—H⋯*A*	*D*—H	H⋯*A*	*D*⋯*A*	*D*—H⋯*A*
O2—H2*B*⋯N3	0.81 (1)	1.89 (3)	2.576 (3)	142 (4)
O4—H4*A*⋯N6	0.82 (1)	1.83 (2)	2.569 (3)	149 (4)
C15—H15⋯O2	0.93	2.55	3.286 (4)	137
C16—H16⋯O1	0.93	2.41	3.185 (4)	141
N2—H2*A*⋯O3^i^	0.85 (1)	2.06 (1)	2.906 (3)	171 (3)
C2—H2⋯O3^i^	0.93	2.52	3.380 (4)	153
N5—H5*A*⋯N1^ii^	0.86 (1)	2.16 (1)	3.009 (4)	170 (3)

Symmetry codes: (i) 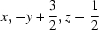; (ii) 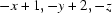.

**Fig. 1.**
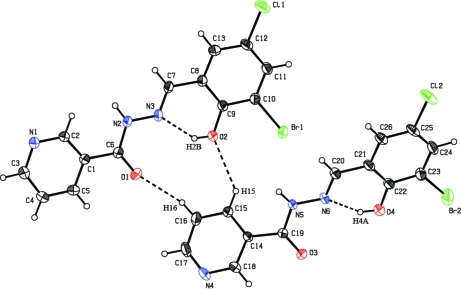
The molecular structure of (I), with atom labels and 30% probability displacement ellipsoids for non-H atoms.

**Fig. 2.**
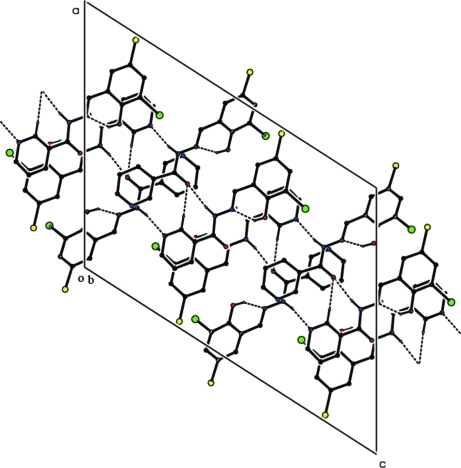
The packing of (I), viewed down b axis. Hydrogen bonds are shown as dashed lines. H atoms not involved in hydrogen bonding have been omitted.

## supplementary crystallographic information

### Comment

The geometric parameters of the title compound (I), agree well with the reported similar structures (Naveenkumar *et al.*, 2010; Su *et al.*, 2010; Tecer *et al.*, 2010). The dihedral angles between the (C1/C2/N1/C3/C4/C5) and (C8-C13) is 8.23 (9)° and (C14/C15/C16/C17/N4/C18) and (C21-C26) is 52.84 (12)°. Both the molecules exist in an E configuration with respect to the C=N double bond. The two molecules in the asymmetric unit are linked via weak C15-H15···O2 and C16-H16···O1 hydrogen bonds. In both the molecules, the intramolecular O2-H2B···N3 and O4-H4A···N6 hydrogen bonds generate six-membered rings, each with graph-set motif S(6) (see, Fig. 1).

In the crystal structure (Fig. 2), the intermolecular N2-H2A···O3 and C2-H2···O3 hydrogen bonds generates a seven-membered ring, with bifurcated R^1^_2_(7) ring motif. The crystal packing is further stabilized due to weak intermolecular N-H···N, N-H···O, C-H···O (Table 1 ) and π···π [Cg4···Cg4 (2-x,1-y,1-z) = 3.614 (2)Å; Cg4 is the centroid of the ring (C21-C26)] interactions.

### Experimental

Nicotinoylhydrazide (5 mmol) was dissolved in 20 mL of dry methanol with stirring and warming over a period of 10 min. To the warm hydrazide solution, 3- bromo-5-chloro salicylaldehyde (5 mmol) in 20 mL of dry methanol was added and the mixture was stirred and slowly refluxed for 2 h. The mixture was then cooled down to room temperature when pale yellow crystalline compound precipitated. The compound was collected by filtration, washed well with cold methanol and dried in vacuum. Single crystals suitable for the X-ray diffraction were obtained by slow evaporation of a solution of the title compound in methanol at room temperature. Melting Point: 503 K.

### Refinement

The amino and hydroxy H-atoms were located in a difference Fourier map, and were refined with N–H distance restraint of 0.86 (1) Å and O-H distance restraint of 0.82 (1) Å. All other H atoms were positioned geometrically with C-H = 0.93 Å, and allowed to ride on their parent atoms, with Uiso(H) = 1.2Ueq(C).

### Crystal data

**Table:**

C_13_H_9_BrClN_3_O_2_	*F*(000) = 1408
*M**_r_* = 354.59	*D*_x_ = 1.736 Mg m^−^^3^
Monoclinic, *P*2_1_/*c*	Mo *K*α radiation, λ = 0.71073 Å
Hall symbol: -P 2ybc	Cell parameters from 7402 reflections
*a* = 18.2217 (5) Å	θ = 2.3–27.8°
*b* = 7.4666 (2) Å	µ = 3.23 mm^−^^1^
*c* = 23.6916 (5) Å	*T* = 295 K
β = 122.685 (1)°	Block, pale yellow
*V* = 2712.93 (12) Å^3^	0.30 × 0.24 × 0.20 mm
*Z* = 8	

### Data collection

**Table:**

Bruker APEXII diffractometer	8116 independent reflections
Radiation source: fine-focus sealed tube	4715 reflections with *I* > 2σ(*I*)
graphite	*R*_int_ = 0.042
ω and φ scans	θ_max_ = 30.4°, θ_min_ = 2.3°
Absorption correction: multi-scan (*SADABS*; Sheldrick, 1996)	*h* = −25→25
*T*_min_ = 0.444, *T*_max_ = 0.564	*k* = −10→10
34991 measured reflections	*l* = −31→33

### Refinement

**Table:**

Refinement on *F*^2^	Primary atom site location: structure-invariant direct methods
Least-squares matrix: full	Secondary atom site location: difference Fourier map
*R*[*F*^2^ > 2σ(*F*^2^)] = 0.047	Hydrogen site location: inferred from neighbouring sites
*wR*(*F*^2^) = 0.125	H atoms treated by a mixture of independent and constrained refinement
*S* = 1.02	*w* = 1/[σ^2^(*F*_o_^2^) + (0.0543*P*)^2^ + 1.4342*P*] where *P* = (*F*_o_^2^ + 2*F*_c_^2^)/3
8116 reflections	(Δ/σ)_max_ < 0.001
377 parameters	Δρ_max_ = 1.27 e Å^−^^3^
4 restraints	Δρ_min_ = −0.84 e Å^−^^3^

### Fractional atomic coordinates and isotropic or equivalent isotropic displacement parameters (Å^2^)

**Table:**

	*x*	*y*	*z*	*U*_iso_*/*U*_eq_	
Br1	0.750800 (19)	0.43505 (5)	0.256817 (14)	0.04545 (11)	
Br2	0.92847 (3)	0.37571 (8)	0.62069 (2)	0.08778 (19)	
Cl1	0.97541 (6)	0.57738 (16)	0.17505 (5)	0.0727 (3)	
Cl2	1.13001 (5)	0.78996 (14)	0.56676 (6)	0.0691 (3)	
O1	0.41709 (16)	0.7827 (4)	−0.01578 (11)	0.0622 (7)	
O2	0.62074 (13)	0.5694 (3)	0.11875 (10)	0.0427 (5)	
O3	0.56100 (13)	0.6644 (3)	0.35290 (10)	0.0471 (6)	
O4	0.78596 (14)	0.5308 (4)	0.49132 (11)	0.0498 (6)	
N1	0.31049 (16)	1.0700 (4)	−0.22503 (12)	0.0412 (6)	
N2	0.50665 (16)	0.7928 (4)	−0.05295 (12)	0.0404 (6)	
N3	0.57128 (15)	0.7165 (4)	0.00520 (11)	0.0390 (6)	
N4	0.34447 (16)	0.7592 (5)	0.16222 (15)	0.0613 (9)	
N5	0.65084 (14)	0.7503 (4)	0.31986 (11)	0.0371 (6)	
N6	0.72089 (14)	0.7123 (4)	0.38238 (11)	0.0386 (6)	
C1	0.36031 (18)	0.9139 (4)	−0.12190 (14)	0.0357 (6)	
C2	0.37331 (18)	0.9923 (4)	−0.16888 (14)	0.0367 (6)	
H2	0.4293	0.9904	−0.1607	0.044*	
C3	0.2310 (2)	1.0706 (5)	−0.23508 (16)	0.0463 (8)	
H3	0.1857	1.1219	−0.2744	0.056*	
C4	0.2119 (2)	1.0005 (5)	−0.19115 (17)	0.0489 (8)	
H4	0.1555	1.0062	−0.2003	0.059*	
C5	0.2773 (2)	0.9218 (5)	−0.13355 (16)	0.0454 (8)	
H5	0.2661	0.8742	−0.1026	0.054*	
C6	0.42963 (19)	0.8249 (4)	−0.05901 (14)	0.0390 (7)	
C7	0.64618 (19)	0.6968 (5)	0.01529 (14)	0.0421 (7)	
H7	0.6568	0.7285	−0.0176	0.051*	
C8	0.71621 (18)	0.6234 (4)	0.07931 (14)	0.0384 (7)	
C9	0.70035 (18)	0.5672 (4)	0.12829 (14)	0.0348 (6)	
C10	0.77050 (19)	0.5086 (4)	0.18998 (14)	0.0367 (6)	
C11	0.85431 (19)	0.5085 (5)	0.20395 (15)	0.0430 (7)	
H11	0.9005	0.4695	0.2454	0.052*	
C12	0.86859 (19)	0.5669 (5)	0.15582 (17)	0.0482 (8)	
C13	0.8007 (2)	0.6208 (5)	0.09373 (17)	0.0478 (8)	
H13	0.8115	0.6557	0.0612	0.057*	
C14	0.49790 (16)	0.7151 (4)	0.23663 (13)	0.0323 (6)	
C15	0.50623 (19)	0.6727 (4)	0.18378 (14)	0.0389 (7)	
H15	0.5603	0.6442	0.1909	0.047*	
C16	0.4322 (2)	0.6739 (5)	0.12011 (15)	0.0513 (9)	
H16	0.4351	0.6456	0.0831	0.062*	
C17	0.3545 (2)	0.7173 (6)	0.11241 (17)	0.0604 (10)	
H17	0.3050	0.7176	0.0691	0.073*	
C18	0.41587 (18)	0.7556 (5)	0.22332 (16)	0.0476 (8)	
H18	0.4106	0.7817	0.2593	0.057*	
C19	0.57123 (17)	0.7085 (4)	0.30792 (13)	0.0340 (6)	
C20	0.79620 (18)	0.7645 (4)	0.39838 (15)	0.0393 (7)	
H20	0.8030	0.8357	0.3693	0.047*	
C21	0.87182 (18)	0.7109 (4)	0.46324 (15)	0.0390 (7)	
C22	0.86279 (18)	0.5946 (4)	0.50526 (15)	0.0404 (7)	
C23	0.9379 (2)	0.5408 (5)	0.56524 (15)	0.0491 (8)	
C24	1.0189 (2)	0.6014 (5)	0.58363 (16)	0.0510 (9)	
H24	1.0682	0.5644	0.6238	0.061*	
C25	1.02624 (18)	0.7172 (5)	0.54212 (17)	0.0473 (8)	
C26	0.95434 (18)	0.7721 (5)	0.48211 (16)	0.0455 (8)	
H26	0.9608	0.8496	0.4543	0.055*	
H2A	0.517 (2)	0.813 (5)	−0.0833 (13)	0.054 (10)*	
H5A	0.655 (2)	0.804 (4)	0.2895 (12)	0.041 (9)*	
H2B	0.5850 (19)	0.618 (5)	0.0836 (11)	0.063 (12)*	
H4A	0.7488 (19)	0.569 (5)	0.4542 (10)	0.068 (13)*	

### Atomic displacement parameters (Å^2^)

**Table:**

	*U*^11^	*U*^22^	*U*^33^	*U*^12^	*U*^13^	*U*^23^
Br1	0.03930 (17)	0.0614 (2)	0.03022 (15)	−0.00234 (15)	0.01524 (12)	0.00653 (14)
Br2	0.0636 (3)	0.1316 (5)	0.0522 (2)	0.0067 (3)	0.0208 (2)	0.0320 (3)
Cl1	0.0330 (4)	0.1031 (8)	0.0796 (7)	0.0043 (5)	0.0287 (4)	0.0239 (6)
Cl2	0.0245 (4)	0.0654 (6)	0.0928 (7)	−0.0062 (4)	0.0157 (4)	−0.0170 (5)
O1	0.0541 (14)	0.102 (2)	0.0400 (13)	0.0159 (14)	0.0314 (11)	0.0236 (13)
O2	0.0291 (10)	0.0656 (15)	0.0317 (11)	0.0023 (10)	0.0152 (9)	0.0069 (11)
O3	0.0347 (11)	0.0793 (17)	0.0298 (10)	−0.0008 (11)	0.0190 (9)	0.0019 (11)
O4	0.0314 (11)	0.0760 (18)	0.0374 (12)	0.0003 (11)	0.0155 (10)	0.0022 (12)
N1	0.0359 (13)	0.0505 (16)	0.0348 (13)	0.0051 (12)	0.0176 (11)	0.0087 (12)
N2	0.0339 (13)	0.0601 (17)	0.0249 (12)	−0.0015 (12)	0.0143 (10)	0.0063 (11)
N3	0.0341 (13)	0.0509 (16)	0.0251 (11)	0.0002 (11)	0.0115 (10)	0.0051 (11)
N4	0.0264 (13)	0.094 (3)	0.0499 (17)	0.0097 (15)	0.0117 (12)	0.0140 (17)
N5	0.0219 (11)	0.0573 (17)	0.0272 (12)	0.0003 (11)	0.0100 (9)	0.0064 (11)
N6	0.0232 (11)	0.0540 (16)	0.0289 (12)	0.0022 (11)	0.0077 (9)	−0.0003 (11)
C1	0.0325 (14)	0.0427 (18)	0.0319 (14)	−0.0003 (13)	0.0174 (12)	−0.0018 (12)
C2	0.0311 (14)	0.0450 (17)	0.0352 (15)	0.0014 (13)	0.0187 (12)	0.0030 (13)
C3	0.0376 (16)	0.055 (2)	0.0391 (16)	0.0046 (15)	0.0160 (13)	0.0046 (15)
C4	0.0322 (15)	0.065 (2)	0.0488 (18)	0.0031 (15)	0.0212 (14)	0.0020 (17)
C5	0.0376 (16)	0.059 (2)	0.0467 (18)	−0.0010 (15)	0.0272 (14)	0.0026 (16)
C6	0.0361 (16)	0.050 (2)	0.0295 (14)	−0.0010 (13)	0.0171 (12)	0.0006 (13)
C7	0.0371 (16)	0.058 (2)	0.0272 (14)	−0.0037 (14)	0.0148 (12)	0.0067 (13)
C8	0.0325 (15)	0.0487 (19)	0.0306 (14)	−0.0015 (13)	0.0149 (12)	0.0043 (13)
C9	0.0294 (13)	0.0389 (16)	0.0317 (13)	−0.0021 (12)	0.0135 (11)	−0.0004 (12)
C10	0.0351 (15)	0.0404 (17)	0.0312 (14)	−0.0037 (13)	0.0156 (12)	0.0011 (12)
C11	0.0305 (15)	0.0516 (19)	0.0368 (16)	−0.0020 (14)	0.0117 (13)	0.0049 (14)
C12	0.0285 (14)	0.062 (2)	0.0513 (19)	−0.0014 (15)	0.0196 (14)	0.0073 (17)
C13	0.0375 (16)	0.065 (2)	0.0461 (18)	−0.0016 (15)	0.0260 (15)	0.0093 (16)
C14	0.0234 (12)	0.0395 (17)	0.0308 (14)	0.0019 (12)	0.0125 (11)	0.0050 (12)
C15	0.0308 (14)	0.054 (2)	0.0307 (14)	−0.0013 (13)	0.0161 (12)	0.0047 (13)
C16	0.0460 (19)	0.070 (2)	0.0288 (15)	−0.0042 (17)	0.0143 (14)	0.0041 (15)
C17	0.0329 (17)	0.087 (3)	0.0378 (18)	−0.0017 (18)	0.0037 (14)	0.0145 (18)
C18	0.0293 (15)	0.067 (2)	0.0448 (18)	0.0084 (15)	0.0190 (14)	0.0077 (16)
C19	0.0279 (13)	0.0443 (17)	0.0304 (14)	0.0024 (12)	0.0162 (11)	−0.0003 (12)
C20	0.0288 (14)	0.0459 (19)	0.0368 (15)	−0.0010 (13)	0.0135 (12)	−0.0013 (13)
C21	0.0248 (13)	0.0464 (18)	0.0373 (15)	0.0014 (13)	0.0112 (12)	−0.0089 (13)
C22	0.0283 (14)	0.054 (2)	0.0342 (15)	0.0028 (13)	0.0137 (12)	−0.0076 (13)
C23	0.0391 (17)	0.065 (2)	0.0342 (15)	0.0096 (16)	0.0142 (13)	−0.0013 (15)
C24	0.0306 (15)	0.065 (2)	0.0368 (16)	0.0095 (15)	0.0047 (13)	−0.0106 (16)
C25	0.0209 (13)	0.051 (2)	0.0547 (19)	−0.0005 (13)	0.0102 (13)	−0.0177 (17)
C26	0.0285 (14)	0.048 (2)	0.0509 (19)	−0.0029 (14)	0.0152 (14)	−0.0088 (15)

### Geometric parameters (Å, °)

**Table:**

Br1—C10	1.885 (3)	C5—H5	0.9300
Br2—C23	1.876 (4)	C7—C8	1.462 (4)
Cl1—C12	1.745 (3)	C7—H7	0.9300
Cl2—C25	1.738 (3)	C8—C13	1.384 (4)
O1—C6	1.205 (4)	C8—C9	1.403 (4)
O2—C9	1.342 (3)	C9—C10	1.394 (4)
O2—H2B	0.814 (10)	C10—C11	1.377 (4)
O3—C19	1.222 (3)	C11—C12	1.370 (5)
O4—C22	1.341 (4)	C11—H11	0.9300
O4—H4A	0.818 (10)	C12—C13	1.375 (4)
N1—C2	1.331 (4)	C13—H13	0.9300
N1—C3	1.336 (4)	C14—C15	1.377 (4)
N2—C6	1.353 (4)	C14—C18	1.384 (4)
N2—N3	1.363 (3)	C14—C19	1.484 (4)
N2—H2A	0.853 (10)	C15—C16	1.378 (4)
N3—C7	1.261 (4)	C15—H15	0.9300
N4—C18	1.325 (4)	C16—C17	1.366 (5)
N4—C17	1.325 (5)	C16—H16	0.9300
N5—C19	1.356 (4)	C17—H17	0.9300
N5—N6	1.364 (3)	C18—H18	0.9300
N5—H5A	0.860 (10)	C20—C21	1.461 (4)
N6—C20	1.272 (4)	C20—H20	0.9300
C1—C5	1.385 (4)	C21—C26	1.394 (4)
C1—C2	1.386 (4)	C21—C22	1.396 (5)
C1—C6	1.492 (4)	C22—C23	1.398 (4)
C2—H2	0.9300	C23—C24	1.371 (5)
C3—C4	1.367 (5)	C24—C25	1.369 (5)
C3—H3	0.9300	C24—H24	0.9300
C4—C5	1.368 (5)	C25—C26	1.376 (4)
C4—H4	0.9300	C26—H26	0.9300
			
C9—O2—H2B	112 (3)	C11—C12—C13	121.2 (3)
C22—O4—H4A	107 (3)	C11—C12—Cl1	118.9 (2)
C2—N1—C3	116.4 (3)	C13—C12—Cl1	119.9 (3)
C6—N2—N3	117.5 (2)	C12—C13—C8	120.4 (3)
C6—N2—H2A	125 (2)	C12—C13—H13	119.8
N3—N2—H2A	117 (2)	C8—C13—H13	119.8
C7—N3—N2	119.8 (2)	C15—C14—C18	118.6 (3)
C18—N4—C17	116.2 (3)	C15—C14—C19	123.6 (2)
C19—N5—N6	116.5 (2)	C18—C14—C19	117.7 (3)
C19—N5—H5A	120 (2)	C14—C15—C16	118.1 (3)
N6—N5—H5A	124 (2)	C14—C15—H15	121.0
C20—N6—N5	119.1 (3)	C16—C15—H15	121.0
C5—C1—C2	117.7 (3)	C17—C16—C15	118.6 (3)
C5—C1—C6	117.6 (3)	C17—C16—H16	120.7
C2—C1—C6	124.8 (3)	C15—C16—H16	120.7
N1—C2—C1	124.0 (3)	N4—C17—C16	124.6 (3)
N1—C2—H2	118.0	N4—C17—H17	117.7
C1—C2—H2	118.0	C16—C17—H17	117.7
N1—C3—C4	124.1 (3)	N4—C18—C14	123.8 (3)
N1—C3—H3	118.0	N4—C18—H18	118.1
C4—C3—H3	118.0	C14—C18—H18	118.1
C3—C4—C5	118.7 (3)	O3—C19—N5	122.0 (3)
C3—C4—H4	120.6	O3—C19—C14	122.3 (3)
C5—C4—H4	120.6	N5—C19—C14	115.7 (2)
C4—C5—C1	119.1 (3)	N6—C20—C21	118.8 (3)
C4—C5—H5	120.4	N6—C20—H20	120.6
C1—C5—H5	120.4	C21—C20—H20	120.6
O1—C6—N2	121.7 (3)	C26—C21—C22	119.8 (3)
O1—C6—C1	121.2 (3)	C26—C21—C20	119.3 (3)
N2—C6—C1	117.1 (2)	C22—C21—C20	120.9 (3)
N3—C7—C8	119.3 (3)	O4—C22—C21	123.7 (3)
N3—C7—H7	120.3	O4—C22—C23	117.8 (3)
C8—C7—H7	120.3	C21—C22—C23	118.4 (3)
C13—C8—C9	119.3 (3)	C24—C23—C22	121.6 (3)
C13—C8—C7	119.5 (3)	C24—C23—Br2	119.0 (2)
C9—C8—C7	121.0 (3)	C22—C23—Br2	119.4 (3)
O2—C9—C10	118.4 (3)	C25—C24—C23	119.1 (3)
O2—C9—C8	122.9 (2)	C25—C24—H24	120.5
C10—C9—C8	118.6 (3)	C23—C24—H24	120.5
C11—C10—C9	121.5 (3)	C24—C25—C26	121.5 (3)
C11—C10—Br1	119.2 (2)	C24—C25—Cl2	117.8 (2)
C9—C10—Br1	119.3 (2)	C26—C25—Cl2	120.7 (3)
C12—C11—C10	119.0 (3)	C25—C26—C21	119.6 (3)
C12—C11—H11	120.5	C25—C26—H26	120.2
C10—C11—H11	120.5	C21—C26—H26	120.2
			
C6—N2—N3—C7	−174.5 (3)	C7—C8—C13—C12	174.4 (3)
C19—N5—N6—C20	−173.2 (3)	C18—C14—C15—C16	0.4 (5)
C3—N1—C2—C1	0.2 (5)	C19—C14—C15—C16	176.2 (3)
C5—C1—C2—N1	−1.8 (5)	C14—C15—C16—C17	0.2 (5)
C6—C1—C2—N1	179.5 (3)	C18—N4—C17—C16	−0.8 (6)
C2—N1—C3—C4	1.3 (5)	C15—C16—C17—N4	0.0 (6)
N1—C3—C4—C5	−1.1 (6)	C17—N4—C18—C14	1.4 (6)
C3—C4—C5—C1	−0.6 (5)	C15—C14—C18—N4	−1.3 (5)
C2—C1—C5—C4	2.0 (5)	C19—C14—C18—N4	−177.4 (3)
C6—C1—C5—C4	−179.2 (3)	N6—N5—C19—O3	10.6 (4)
N3—N2—C6—O1	−2.4 (5)	N6—N5—C19—C14	−167.8 (3)
N3—N2—C6—C1	178.0 (3)	C15—C14—C19—O3	−145.3 (3)
C5—C1—C6—O1	−9.7 (5)	C18—C14—C19—O3	30.6 (5)
C2—C1—C6—O1	169.1 (3)	C15—C14—C19—N5	33.1 (4)
C5—C1—C6—N2	169.9 (3)	C18—C14—C19—N5	−151.0 (3)
C2—C1—C6—N2	−11.3 (5)	N5—N6—C20—C21	−175.0 (3)
N2—N3—C7—C8	176.8 (3)	N6—C20—C21—C26	−178.0 (3)
N3—C7—C8—C13	−172.5 (3)	N6—C20—C21—C22	4.2 (5)
N3—C7—C8—C9	3.0 (5)	C26—C21—C22—O4	179.3 (3)
C13—C8—C9—O2	178.5 (3)	C20—C21—C22—O4	−2.9 (5)
C7—C8—C9—O2	3.0 (5)	C26—C21—C22—C23	−0.9 (5)
C13—C8—C9—C10	−0.6 (5)	C20—C21—C22—C23	176.9 (3)
C7—C8—C9—C10	−176.1 (3)	O4—C22—C23—C24	−179.3 (3)
O2—C9—C10—C11	−177.8 (3)	C21—C22—C23—C24	0.9 (5)
C8—C9—C10—C11	1.3 (5)	O4—C22—C23—Br2	2.9 (4)
O2—C9—C10—Br1	0.1 (4)	C21—C22—C23—Br2	−176.9 (2)
C8—C9—C10—Br1	179.3 (2)	C22—C23—C24—C25	−0.1 (5)
C9—C10—C11—C12	−0.2 (5)	Br2—C23—C24—C25	177.8 (3)
Br1—C10—C11—C12	−178.2 (3)	C23—C24—C25—C26	−0.8 (5)
C10—C11—C12—C13	−1.6 (5)	C23—C24—C25—Cl2	−179.6 (3)
C10—C11—C12—Cl1	176.8 (3)	C24—C25—C26—C21	0.9 (5)
C11—C12—C13—C8	2.4 (6)	Cl2—C25—C26—C21	179.6 (2)
Cl1—C12—C13—C8	−176.0 (3)	C22—C21—C26—C25	0.0 (5)
C9—C8—C13—C12	−1.2 (5)	C20—C21—C26—C25	−177.8 (3)

### Hydrogen-bond geometry (Å, °)

**Table:**

*D*—H···*A*	*D*—H	H···*A*	*D*···*A*	*D*—H···*A*
O2—H2B···N3	0.81 (1)	1.89 (3)	2.576 (3)	142 (4)
O4—H4A···N6	0.82 (1)	1.83 (2)	2.569 (3)	149 (4)
C15—H15···O2	0.93	2.55	3.286 (4)	137
C16—H16···O1	0.93	2.41	3.185 (4)	141
N2—H2A···O3^i^	0.85 (1)	2.06 (1)	2.906 (3)	171 (3)
C2—H2···O3^i^	0.93	2.52	3.380 (4)	153
N5—H5A···N1^ii^	0.86 (1)	2.16 (1)	3.009 (4)	170 (3)

Symmetry codes: (i) *x*, −*y*+3/2, *z*−1/2; (ii) −*x*+1, −*y*+2, −*z*.
